# Association between preoperative proton pump inhibitor use and postoperative acute kidney injury in patients undergoing major surgery

**DOI:** 10.1080/0886022X.2024.2379596

**Published:** 2024-08-04

**Authors:** Xizi Zheng, Qingqing Zhou, Yidan Zhu, Lingyi Xu, Damin Xu, Jicheng Lv, Li Yang

**Affiliations:** a Renal Division, Department of Medicine, Institute of Nephrology, Peking University First Hospital, Peking University, Beijing, China; b China Key Laboratory of Renal Disease, Ministry of Health of China, Beijing, China; c Key Laboratory of CKD Prevention and Treatment, Ministry of Education of China, Beijing, China; d Research Units of Diagnosis and Treatment of Immune-Mediated Kidney Diseases, Chinese Academy of Medical Sciences, Beijing, China; e Clinical Research Institute, Institute of Advanced Clinical Medicine, Peking University, Beijing, China

**Keywords:** Proton pump inhibitor, acute kidney injury, major surgery, nephrotoxicity, mortality

## Abstract

**Background:**

Acute kidney injury (AKI) is a severe postoperative complication in patients undergoing major surgery. Proton pump inhibitors (PPIs) are used preoperatively as prophylaxis for postoperative gastrointestinal bleeding. Whether preoperative PPI use is associated with an increased risk of postoperative AKI remains uncertain.

**Methods:**

This retrospective cohort study used electronic medical records from the clinical data warehouse of Peking University First Hospital to screen all adult hospitalizations undergoing major surgery between 1 January 2018 and 31 December 2020. Exposure was preoperative PPI use, defined as PPI use within 7 days before major surgery. The primary outcome was postoperative AKI, defined as AKI occurring within 7 days after major surgery; secondary outcomes included in-hospital AKI and in-hospital mortality.

**Results:**

A total of 21,533 patients were included in the study (mean [*SD*] age, 57.8 [15.0] years; 51.2% male), of which 944 (4.4%) were prescribed PPI within 7 days before major surgery (PPI users). Overall, 72 PPI users (7.6%) and 356 non-users (1.7%) developed postoperative AKI. After adjustment, preoperative PPI use was associated with an increased risk of postoperative AKI (adjusted OR, 1.47; 95% CI, 1.04–2.07) and in-hospital AKI (adjusted OR, 1.41; 95% CI, 1.03–1.94). Moreover, subgroup analyses showed that the risk of PPI on postoperative AKI was amplified by the concomitant use of non-steroidal anti-inflammatory drugs or diuretics. No significant difference was observed between preoperative PPI use and in-hospital mortality in the fully adjusted model (adjusted OR 1.63; 95% CI, 0.55–4.85).

**Conclusions:**

Preoperative PPI use was associated with an increased risk of AKI in patients undergoing major surgery. This risk may be enhanced by the concomitant use of other nephrotoxic drugs. Clinicians should weigh the pros and cons before initiating PPI prophylaxis.

## Introduction

Acute kidney injury (AKI) is a frequent and severe postoperative complication in patients undergoing major surgery, with an incidence varying from 5.3 to 18.4% [1,2]. Postoperative AKI is associated with higher mortality rates, more extended hospital stays, and increased healthcare costs; surviving patients were also at a higher risk of developing chronic kidney disease (CKD) in the long term [1–5].

Proton pump inhibitors (PPIs), a powerful acid-suppressive medication, are widely used in the treatment and prevention of acid-related disorders. Despite being perceived as a well-tolerated class of drugs with a good safety profile, the continuous increase in PPI use has raised concerns about its side effects on the kidneys [6–11]. A growing number of cases reported that long-term PPI use might result in acute interstitial nephritis, a common cause of AKI [6,12–14]. Associations between PPIs and increased risk of AKI have been described among the general population [7,15,16], elderly patients [17], and those with rheumatoid arthritis [18].

Prophylactic short-term PPI therapy is recommended in surgical patients at high risk of gastrointestinal bleeding, such as those with a previous history of peptic ulcer or gastrointestinal bleeding [19,20]. Yet, the kidney safety of PPI prophylaxis is seldom mentioned. Only a few studies limited to patients with cardiac surgery have explored the association between preoperative PPI use and postoperative AKI, and the results did not reach an agreement [21–23]. Based on the elusive evidence, we intended to examine whether preoperative PPI use is associated with an increased risk of AKI in patients undergoing major surgery, with postoperative AKI as the primary outcome. Secondary outcomes included in-hospital AKI and in-hospital mortality.

## Materials and methods

### Study design and population

**Figure 1.**
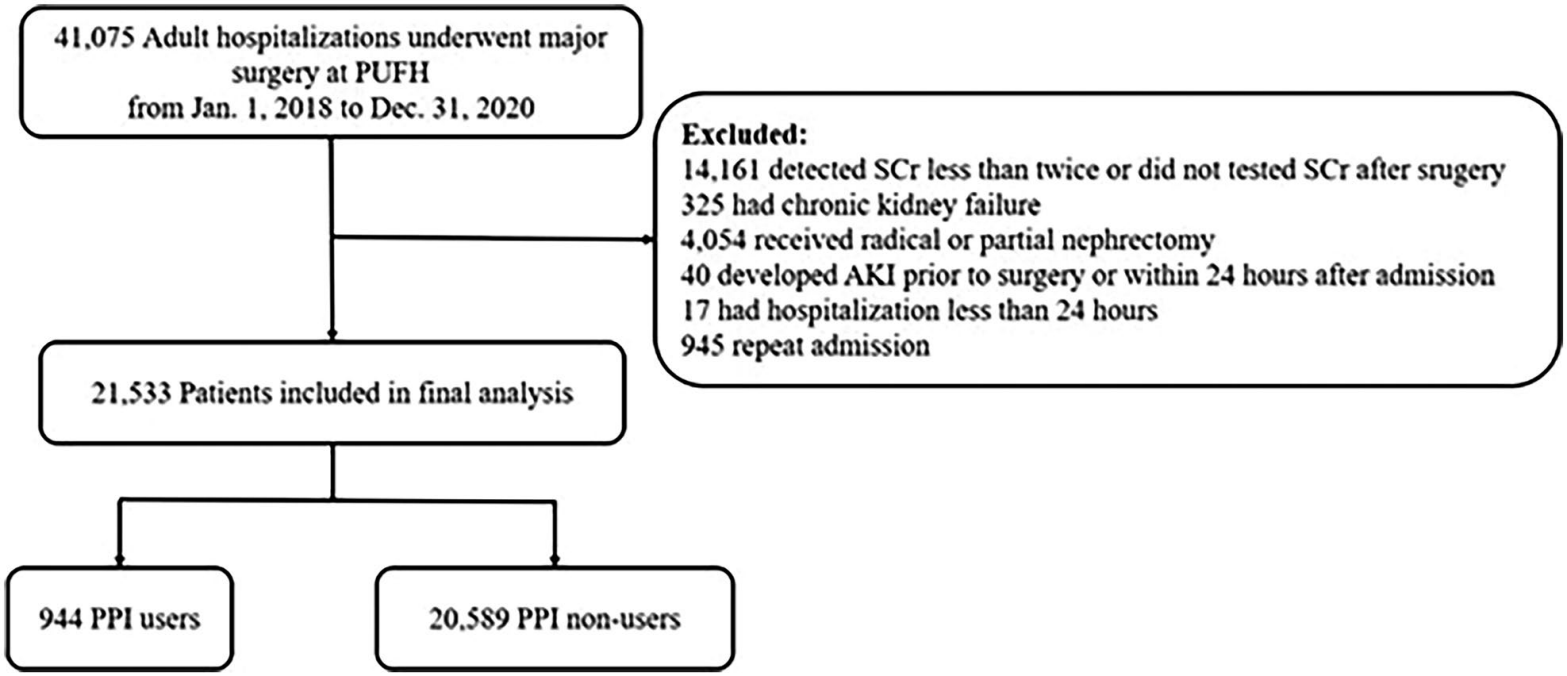
Flowchart of study participants.

This retrospective cohort study was conducted at the Peking University First Hospital (PUFH), China. All adult hospitalizations (≥18 years old) receiving major surgery between 1 January 2018 and 31 December 2020 were included. Major surgery was defined as any surgical procedure under general anesthesia in an operating room [24]. Patients were excluded if they (1) had serum creatinine (SCr) detected less than twice during the hospital stay, or did not have SCr test after surgery; (2) had chronic kidney disease G5 (including long-term dialysis, kidney transplantation prior or current, and baseline estimated glomerular filtration rate [eGFR] < 15 mL/min/1.73 m^2^); (3) received radical or partial nephrectomy; (4) developed AKI before surgery or within 24 h after admission (with admission diagnosis of AKI, or met the criteria of AKI definition within 24 h after admission); (5) had hospital stay <24 h. If participants had more than one qualifying hospitalization during the episode, only the first one was included in the study. The flowchart of the study is presented in Figure 1. The ethical approval was granted by the Clinical Research Ethics Committee of Peking University First Hospital (2023-833), and the requirement of informed consent was waived because of the retrospective nature of this study.

### Data collection

Data during a hospital stay were obtained from the electronic medical records in the clinical data warehouse of PUFH. The baseline was defined as the episode from the admission date to the surgery date or the first surgery date for patients who underwent more than one surgery during one hospitalization. Baseline information included demographic information, chronic comorbidities, laboratory tests, concomitant medication use before surgery, and surgery profiles. Chronic comorbidities were identified by the admitting diagnoses according to the International Classification of Diseases, Tenth Edition (ICD-10). The baseline laboratory value was defined as the first value executed after admission before surgery. Baseline eGFR was calculated using the CKD Epidemiology Collaboration (CKD-EPI) equation that showed good accuracy when estimating GFR in Chinese [25,26]. Medication use before surgery was determined according to prescription records before major surgery during hospitalization. Information related to the risk of gastrointestinal bleeding was collected. Surgery profiles were extracted from the operation and anesthesia management system. Participants were followed up until discharge or a maximum period of up to 30 days after surgery, and data on mortality and AKI were collected during follow-up.

### Exposure assessment

PPI use was identified according to prescription information from the clinical data warehouse. As PPI for gastrointestinal bleeding or stress ulcer prophylaxis is usually used preoperatively for a short period, we defined preoperative PPI use as a prescription within 7 days before major surgery. Patients were divided into PPI users and non-users according to whether they had a PPI prescription within 7 days before surgery.

PPI users were further divided into intravenous group and oral group according to the administrative routes. Those received both intravenous and oral routes were classified as intravenous group. We initially intended to explore the association between PPI dosage and postoperative AKI, however, 91.8% PPI users were prescribed with standard dose [27]. There is no sufficient sample size to support the analysis, as only 5 AKI events were detected in the high dose group (5/77).

### Outcomes

The primary outcome was postoperative AKI, defined as AKI developed within 7 days after major surgery. We detected AKI based on the change of SCr level in accordance with 2012 Kidney Disease: Improving Global Outcomes (KDIGO) AKI definition: an increase in SCr either by 50% within 7 days or more than 26.5 μmol/L within 48 h [28].

Secondary outcomes included in-hospital AKI and in-hospital mortality. In-hospital AKI was defined as AKI occurring after major surgery before the end of follow-up. In-hospital mortality was defined as death until discharge or within a maximum period of up to 30 days after surgery.

We initially set gastrointestinal bleeding as one of the secondary outcomes. However, given that only 12 gastrointestinal bleeding events were detected (3 in PPI users, and 9 in PPI non-users), there was insufficient statistical power to explore this relationship in the current analysis.

### Covariates

Covariates at or before major surgery were chosen based on the potential to confound the relationship between preoperative PPI use and clinical outcomes, including demographic parameters (age, gender, and body mass index [BMI]), chronic comorbidities (hypertension, diabetes mellitus, cardiovascular disease, cerebrovascular disease, peripheral vascular disease, and chronic obstructive pulmonary disease), baseline laboratory tests (eGFR, and hemoglobin), risk of gastrointestinal bleeding (previous history of peptic ulcer or gastrointestinal bleeding, and coagulopathy), medication use before surgery (antibiotics, renin-angiotensin-aldosterone system inhibitor [RAASi], non-steroidal anti-inflammatory drugs [NSAIDs], diuretics, and contrast), and surgery profiles (cardiac surgery, emergent surgery, laparoscopy, American Society of Anesthesiologists [ASA] score ≥3, duration of surgery ≥120 min, and intraoperative blood infusion). Coagulopathy was defined as any of the following: (1) baseline platelet count <50 × 10^9^/L, (2) baseline prothrombin time/international normalization ratio (PT/INR) >1.5, (3) baseline partial-prothrombin time >2 times the control value [29,30]. Antibiotics use refers to any prescription of commonly used preoperative prophylactic antibiotics, including β-lactam, quinolone, and macrolide.

### Statistical analyses

Categorical variables were presented by frequencies (percentages), while quantitative variables were described as mean (standard deviation, *SD*) or median (interquartile range, IQR). Comparisons between PPI users and non-users were made by using Student’s *t*-test, Wilcoxon rank test, or chi-square test.

We used a logistic regression model to explore the association between preoperative PPI use and outcomes. Crude association between PPI use and postoperative AKI was first tested in the unadjusted model. We further built a multivariable logistic regression model adjusted for prespecified covariates including demographic parameters, chronic comorbidities, baseline laboratory tests, risk of gastrointestinal bleeding, medication use before surgery, and surgery profiles. Crude and adjusted odds ratios (ORs) with corresponding 95% confidence intervals (CIs) were reported. Multiple imputation was used to deal with missing data in multivariable analysis.

To assess effect modification, preplanned subgroup analyses were conducted in patients stratified by age (≤65 *vs.* >65 years), eGFR < 60 mL/(min·1.73 m^2^) (yes *vs.* no), duration of surgery (≥120 *vs.* <120 min), antibiotics use (yes *vs.* no), NSAIDs use (yes *vs.* no), RAASi use (yes *vs.* no), and diuretics use (yes *vs.* no). The risk of postoperative AKI was also compared between PPI users with different administrative routes.

Three sensitivity analyses were performed to evaluate the robustness of the results. We first conducted a 1:1 propensity score matching (PSM) analysis. Each PPI user was matched to one PPI non-user across prespecified covariates described above, using a simple nearest neighbor approach with no replacement and a caliper width of 0.2. The balance in baseline covariates between the two groups was assessed using the standardized mean difference (SMD). Second, for the primary outcome analysis, we eliminated patients with long-term PPI indications, including those with gastroesophageal reflux disease (GERD), Barrett esophagus, upper gastrointestinal bleeding, peptic ulcer, severe esophagitis, Helicobacter Pylori (HP) eradication [27,31,32], because this study aimed to evaluate the effect of relatively short-term use of PPI before surgery. Third, we excluded those who initiated PPI use after surgery to eliminate the effect of postoperative PPI use.

The data were analyzed and managed with SPSS Statistics (version 26) and R (version 4.3.2) within the R studio platform (version 2023.12.0 + 369). All *p*-values were two-sided, and *p* < 0.05 was considered statistically significant.

## Results

### Demographic and clinical characteristics

A total of 21,533 patients were included in the study (Figure 1), of which 944 (4.4%) received PPI before major surgery (PPI users). As shown in Table 1, the mean age of the study population was 57.8 (±15.0) years, and 51.2% were male. Compared with non-users, PPI users were older, more likely to suffer from all the chronic comorbidities we compared, and were more likely to be at risk of gastrointestinal bleeding. Likewise, more frequent use of concomitant medications, and higher rates of cardiac and emergent surgery were observed in PPI users.

**Table 1.** Demographic and clinical characteristics of PPI users and Non-users.

Variable	All (*n* = 21,533)	PPI users (*n* = 944)	PPI non-users (*n* = 20,589)	*p*-Value
Demographic				
Age, years, mean (*SD*)	57.8 (15.0)	62.5 (13.9)	57.6 (15.0)	<0.001
Male gender, *n* (%)	11,034 (51.2)	514 (54.4)	10,520 (51.1)	0.046
BMI, kg/m^2^, mean (*SD*)	24.5 (3.8)	23.7 (4.0)	24.5 (3.7)	<0.001
Chronic comorbidities				
Hypertension, *n* (%)	7411 (34.4)	395 (41.8)	7016 (34.1)	<0.001
Diabetes mellitus, *n* (%)	3427 (15.9)	194 (20.6)	3233 (15.7)	<0.001
Cardiovascular disease, *n* (%)	2086 (9.7)	201 (21.3)	1885 (9.2)	<0.001
Cerebrovascular disease, *n* (%)	1230 (5.7)	125 (13.2)	1105 (5.4)	<0.001
Peripheral vascular disease, *n* (%)	801 (3.7)	52 (5.5)	749 (3.6)	0.003
Chronic obstructive pulmonary disease, *n* (%)	682 (3.2)	46 (4.9)	636 (3.1)	0.002
Baseline laboratory tests				
eGFR, ml/(min·1.73 m^2^), mean (*SD*)	92.4 (18.5)	88.6 (19.6)	92.6 (18.5)	<0.001
eGFR < 60 ml/(min·1.73 m^2^), *n* (%)	1203 (5.7)	86 (9.4)	1117 (5.6)	<0.001
Hemoglobin, g/L, mean (*SD*)	134.9 (17.9)	125.4 (22.2)	135.4 (17.6)	<0.001
Platelet, 10^9^/L, mean (*SD*)	226.0 (69.4)	225.8 (80.2)	226.0 (68.8)	0.922
Serum albumin, g/L, mean (*SD*)	42.0 (5.2)	39.3 (5.4)	42.2 (5.1)	<0.001
Risk of gastrointestinal bleeding				
Previous history of peptic ulcer or gastrointestinal bleeding, *n* (%)	195 (0.9)	65 (6.9)	130 (0.6)	<0.001
Coagulopathy*, *n* (%)	125 (0.6)	15 (1.6)	110 (0.5)	<0.001
Medication use before surgery				
Antibiotics, *n* (%)	14,224 (66.1)	857 (90.8)	13,367 (64.9)	<0.001
RAASi, *n* (%)	947 (4.4)	125 (13.2)	822 (4.0)	<0.001
NSAIDs, *n* (%)	1015 (4.7)	346 (36.7)	669 (3.2)	<0.001
Diuretics, *n* (%)	584 (2.7)	148 (15.7)	436 (2.1)	<0.001
Glucocorticoid, *n* (%)	935 (4.3)	139 (14.7)	796 (3.9)	<0.001
Contrast, *n* (%)	357 (1.7)	63 (6.7)	294 (1.4)	<0.001
Anticoagulant, *n* (%)	3212 (14.9)	437 (46.3)	2775 (13.5)	<0.001
H2-receptor antagonist, *n* (%)	29 (0.1)	4 (0.4)	25 (0.1)	0.036
Statin, *n* (%)	931 (4.3)	182 (19.3)	749 (3.6)	<0.001
Surgery profiles				
Cardiac, *n* (%)	288 (1.3)	41 (4.3)	247 (1.2)	<0.001
Aortic, *n* (%)	54 (0.3)	2 (0.2)	52 (0.3)	0.807
Laparoscopy, *n* (%)	5899 (27.4)	196 (20.8)	5703 (27.7)	<0.001
Emergent, *n* (%)	538 (2.5)	99 (10.5)	439 (2.1)	<0.001
ASA score ≥3, *n* (%)	4271 (19.8)	397 (42.1)	3874 (18.8)	<0.001
Duration of surgery ≥120 min, *n* (%)	17,284 (80.3)	869 (92.1)	16,415 (79.7)	<0.001
Intraoperative blood transfusion, *n* (%)	1742 (8.1)	157 (16.6)	1585 (7.7)	<0.001

BMI: body mass index; eGFR: estimated glomerular filtration rate; RAASi: renin-angiotensin-aldosterone system inhibitor; NSAIDs: non-steroidal anti-inflammatory drugs; ASA: American Society of Anesthesiologists.

Missing values (*n*, %): BMI (559, 2.6%), baseline eGFR (523, 2.4%), baseline albumin (1889, 8.8%), baseline hemoglobin (506, 2.3%), baseline platelet (729, 3.4%). Multiple imputation was used to impute missing values.

* Coagulopathy was defined as any of the following: (1) baseline platelet count <50 × 10^9^/L, (2) baseline prothrombin time/international normalization ratio (PT/INR) >1.5, (3) baseline partial-prothrombin time >2 times the control value.

### Association between preoperative PPI use and postoperative AKI

Overall, 72 of 944 PPI users (7.6%) and 356 of 20,589 non-users (1.7%) developed postoperative AKI (Table 2). In the unadjusted model, preoperative PPI use was associated with a statistically significant increased risk of postoperative AKI (crude OR, 4.69; 95% CI, 3.61–6.10). The association was attenuated but still remained significant in the multivariate logistic regression model after adjusting for potential confounders (adjusted OR 1.47; 95% CI, 1.04–2.07) (Table 2).

**Table 2.** Association of PPI use and clinical outcomes in patients undergoing major surgery.

Outcomes	No. patients with event, *n* (%)		Odds ratio (95% confidence interval)	
	PPI users, *n* (%)	PPI non-users, *n* (%)	Crude	Adjusted^a^
Primary				
Postoperative AKI	72 (7.6)	356 (1.7)	4.69 (3.61, 6.10)	1.47 (1.04, 2.07)
Secondary				
In-hospital AKI	85 (9.0)	442 (2.1)	4.51 (3.54, 5.75)	1.41 (1.03, 1.94)
In-hospital mortality	9 (1.0)	19 (0.1)	10.42 (4.70, 23.10)	1.63 (0.55, 4.85)

^a^ Adjusted for demographic parameters (age, gender, and BMI), chronic comorbidities (hypertension, diabetes mellitus, cardiovascular disease, cerebrovascular disease, peripheral vascular disease, and chronic obstructive pulmonary disease), baseline laboratory tests (eGFR, and hemoglobin), risk of gastrointestinal bleeding (previous history of peptic ulcer or gastrointestinal bleeding, and coagulopathy), medication use before surgery (antibiotics, RAASi, NSAIDs, diuretics, and contrast), and surgery profiles (cardiac surgery, laparoscopy, emergent surgery, ASA score ≥3, duration of surgery ≥120 min, and intraoperative blood infusion).

**Figure 2.**
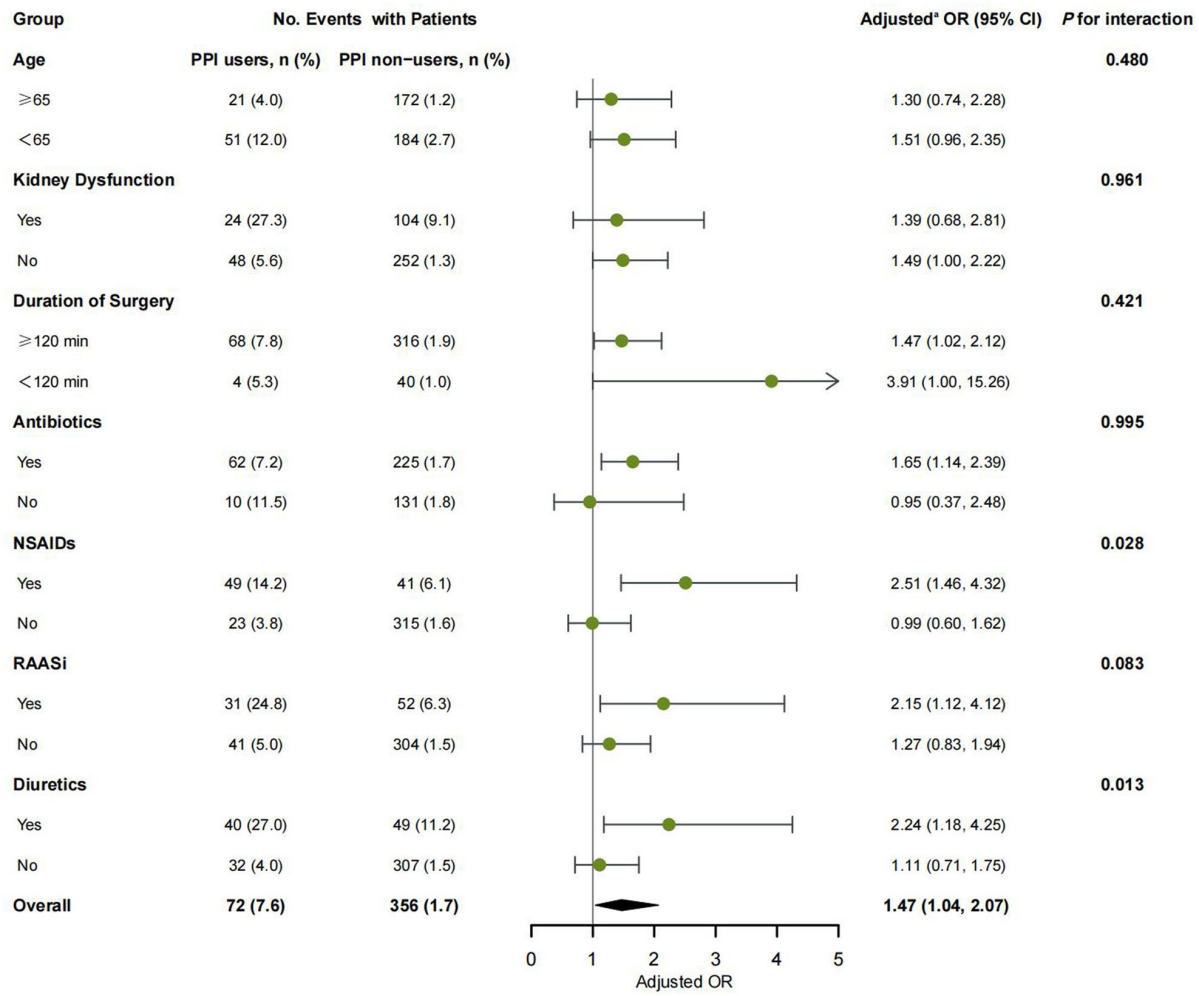
Subgroup analyses of postoperative AKI stratified by age, eGFR, and medication use before surgery. ^a^Adjusted for demographic parameters (age, gender, and BMI), chronic comorbidities (hypertension, diabetes mellitus, cardiovascular disease, cerebrovascular disease, peripheral vascular disease, and chronic obstructive pulmonary disease), baseline laboratory tests (eGFR, and hemoglobin), risk of gastrointestinal bleeding (previous history of peptic ulcer or gastrointestinal bleeding, and coagulopathy), medication use before surgery (antibiotics, RAASi, NSAIDs, diuretics, and contrast), and surgery profiles (cardiac surgery, laparoscopy, emergent surgery, ASA score ≥3, duration of surgery ≥120 min, and intraoperative blood infusion).

In subgroup analysis, a significant interaction was observed between preoperative PPI and NSAIDs use (*p* for interaction = 0.028). PPI use was related to a statistically significant increased risk of postoperative AKI in patients with concomitant use of NSAIDs (adjusted OR, 2.51; 95% CI, 1.46–4.32) compared with those without NSAIDs (adjusted OR, 0.99; 95% CI, 0.60–1.62). A similar effect was observed between preoperative PPI and diuretics use, as the association of preoperative PPI use and postoperative AKI was more significant in patients with concomitant use of diuretics (adjusted OR, 2.24; 95% CI, 1.18–4.25) compared with those without diuretics (adjusted OR, 1.11; 95% CI, 0.71–1.75; *p* for interaction = 0.013). No significant interaction was observed in subgroups stratified by age, eGFR, duration of surgery, antibiotics use, and RAASi use (all *p* for interaction >0.05) (Figure 2).

Among PPI users, 681 patients (72.1%) received intravenous PPI, whereas 263 patients (27.9%) received oral PPI. Postoperative AKI occurred in 42 intravenous PPI users (6.2%) and 30 oral PPI users (11.4%). Compared with non-users, both oral (crude OR, 7.32; 95% CI 4.93–10.86) and intravenous PPI (crude OR, 3.74; 95% CI 2.69–5.19) were associated with an increased risk of postoperative AKI. After adjustment, this association stayed significant in intravenous PPI users (adjusted OR 1.82; 95% CI 1.19–2.78), yet it was attenuated and did not remain significant in oral PPI users (adjusted OR 1.20; 95% CI 0.73–1.97).

### Association between preoperative PPI use and in-hospital AKI

During a median of 5.8 days of follow-up, a total of 527 patients (2.4%) developed in-hospital AKI, of which 85 were PPI users (9.0%) and 442 were non-users (2.1%). A significant association between PPI use and in-hospital AKI was also observed in the unadjusted model (crude OR, 4.51; 95% CI, 3.54–5.75), and the association stayed significant in the adjusted model (adjusted OR, 1.41; 95% CI, 1.03–1.94) (Table 2).

### Association between PPI use and in-hospital mortality

During a median of 5.8 days of follow-up, 28 patients died, of which 9 were PPI users (1.0%) and 19 were non-users (0.1%). PPI use was associated with a remarkably increased risk of in-hospital mortality in the unadjusted model (crude OR, 10.42; 95% CI, 4.70–23.10), yet the association turned out to be insignificant in the fully adjusted model (adjusted OR, 1.63; 95% CI, 0.55–4.85) (Table 2).

### Sensitivity analyses

In PSM analysis, 902 of 944 PPI users (95.6%) were 1:1 matched to similar patients without PPI use (Supplementary Table 1). Two groups in the PSM cohort were well-balanced across all included covariates (all standardized mean differences <10%). Consistent with the results of the multivariate logistic regression, PPI users had higher rates of postoperative AKI (6.9 *vs.* 4.2%, *p* = 0.018) and in-hospital AKI (8.2 *vs.* 5.0%, *p* = 0.008) compared with non-users. No significant difference was found in in-hospital mortality between the two groups (0.7 *vs.* 0.2%, *p* = 0.288).

The association between preoperative PPI use and postoperative AKI remains significant when excluding patients with long-term PPI indications (adjusted OR, 1.61; 95% CI, 1.12–2.32) or in the population that excluded those initiated PPI after surgery (adjusted OR, 1.75; 95% CI, 1.06–2.89) (Supplementary Table 2).

## Discussion

In this retrospective cohort study, we analyzed clinical data of 21,533 patients undergoing major surgery to assess the association between preoperative PPI use and the risk of postoperative AKI. After adjusting for confounding variables, preoperative PPI use was associated with an increased risk of postoperative AKI. This association remained statistically significant in a well-balanced PSM analysis and several other sensitivity analyses. Furthermore, significant positive interactions between PPI and other nephrotoxic drugs (NSAIDs and diuretics) were also observed.

PPIs are among the most commonly prescribed drugs in the world. Recently, emerging evidence has highlighted the side effects of PPI use, including increased risk of AKI [7,17,18,33]. In a population-based study involving more than 290,000 PPI users and non-users aged 66 years and older, PPI use significantly increased the risk of AKI (hazard ratio [HR], 2.52; 95% CI, 2.27–2.79) [17]. Similarly, in a recent meta-analysis including 2,492,125 individuals from 12 observational studies, a significant positive correlation between PPI use and the risk of AKI was also observed (adjusted relative risk [RR] 1.75; 95% CI, 1.40–2.19) [33]. Several mechanisms might be involved, with acute interstitial nephritis (AIN) being proposed as the main cause [13,34]. PPIs and their metabolites could induce an immune response in different ways, leading to AIN [6]. In addition, PPIs could increase oxidative stress, affect the function of mitochondria and lysosomes in renal tubular cells, and finally lead to cell death and kidney damage [35]. The immune response to dying cells could result in interstitial inflammation [35]. Moreover, endothelial dysfunction and vasoconstriction induced by oxidative stress [36] may also contribute to the development of AKI.

Prophylactic PPI therapy was recommended in surgical patients at high risk of gastrointestinal bleeding [37–42]. Limited studies explored the association between preoperative PPI use and AKI, and the results did not reach an agreement. It was worth mentioning that PPI was commonly identified by pre-admission prescription in these studies, which might reflect a relatively long-term exposure [7,17,34,43]. Acute interstitial nephritis, the leading cause of AKI, was reported to occur after an average of 11 weeks of PPI initiation [44]. However, for gastrointestinal bleeding prophylaxis, PPIs were usually prescribed for a short period perioperatively and were discontinued when the clinical condition improved [37–42]. A previous study found that PPI use within 3 weeks before cardiac surgery was related to an increased risk of postoperative AKI, yet the risk was not elevated when PPI was discontinued preoperatively [21]. In this study, we defined preoperative PPI use as prescriptions within one week before surgery, the most common period for prophylaxis, and found preoperative PPI use was associated with an increased risk of postoperative AKI. This association was enhanced when we further excluded patients with indications for long-term PPI use. It is worth mentioning that the inappropriate prophylactic use of PPI during the perioperative period has become a rising issue [45,46]. This prophylactic use was found to be less effective in patients at low risk for gastrointestinal bleeding [47–49]. In this study, only 1.5% of PPI users patients were at high risk, and limited bleeding events were observed. Our findings indicated clinicians should balance the risk of AKI with the reward of bleeding prophylaxis to avoid inappropriate PPI overuse in clinical practice.

In addition, drug interactions affecting AKI were observed when PPIs were used together with NSAIDs or diuretics, which can alter the kidney hemodynamic autoregulation system, constrict the blood flow into the glomerulus, and lead to kidney ischemia and hypoxia [50–52]. Concurrent use of these nephrotoxic drugs might act as a double or triple whammy to the kidney and lead to AKI [51–53]. It is worth mentioning that patients with NSAIDs exposure also have an increased risk of gastrointestinal bleeding and, therefore, are more likely to receive PPIs [31]. In those with strong indications for PPI use, avoidance of other nephrotoxins should be considered, and timely discontinuation should be facilitated after the perioperative period, as the increased risk of AKI was alleviated when PPI was discontinued [21,34].

This study has several limitations. First, due to the lack of outpatient data, the effect of preadmission PPI use could not be ascertained. We further excluded patients with indications for long-term PPI use at admission in sensitivity analysis to attenuate this effect. Second, due to the observational nature of the study, causality cannot be determined on the current design; we performed the multivariable adjustment and PSM analysis to lower the chances of bias, and the findings remained consistent. Third, although we’ve set several criteria to exclude patients with preoperative AKI, a few cases might be included due to a lack of enough SCr tests. Fourth, because urine output was unavailable in this study, some patients with AKI might remained undetected. Fifth, some possible cofounders, such as sepsis, hemodilution, and intraoperative hypotension could not be obtained, which might result in bias. Finally, the benefit of PPI use on postoperative gastrointestinal bleeding could not be evaluated due to insufficient bleeding events. Prospective studies should be conducted to verify the potential nephrotoxicity of PPI in the future.

## Conclusions

In this study, preoperative PPI use was associated with an increased risk of AKI in patients undergoing major surgery. This risk was enhanced by concomitant therapy of NSAIDs and diuretics. Therefore, clinicians should weigh the pros and cons before initiating PPI prophylaxis in patients undergoing major surgery.

## Supplementary Material

Supplementary new.docx

## Data Availability

The datasets are not publicly available as they are protected by Peking University First Hospital. Access requests should be directed to the corresponding author.
