# Supplementary material for: Association between preoperative proton pump inhibitor use and postoperative acute kidney injury in patients undergoing major surgery
Source: Ren Fail. 2024 Aug 4;46(2):2379596. doi: 10.1080/0886022X.2024.2379596 (PMC11302470; doi:10.1080/0886022X.2024.2379596)
Supplement: Supplementary new.docx [file IRNF_A_2379596_SM5312.docx]

**Supplementary Table 1. Comparison of Baseline Characteristics and Clinical Outcomes between PPI Users and Non-users after Propensity Score Matching**

|  | **PPI users** | **PPI non-users** | **SMD, %** | ***P*-value** |
| --- | --- | --- | --- | --- |
|  | **(n=902)** | **(n=902)** |  |  |
| **Demographic** |  |  |  |  |
| Age, years, mean (SD) | 62.2 (13.9) | 61.5 (14.8) | 4.76 | 0.326 |
| Male Gender, n (%) | 491 (54.4) | 485 (53.8) | 1.34 | 0.813 |
| BMI, kg/m^2^, mean (SD) | 23.8 (4.0) | 23.9 (3.7) | 4.89 | 0.454 |
| **Chronic Comorbidities** |  |  |  |  |
| Hypertension, n (%) | 374 (41.5) | 376 (41.7) | 0.45 | 0.962 |
| Diabetes Mellitus, n (%) | 184 (20.4) | 187 (20.7) | 0.82 | 0.907 |
| Cardiovascular Disease, n (%) | 181 (20.1) | 176 (19.5) | 1.35 | 0.813 |
| Cerebrovascular Disease, n (%) | 112(12.4) | 111 (12.3) | 0.33 | 1.000 |
| **Baseline Laboratory Tests** |  |  |  |  |
| eGFR<60ml/(min·1.73m2), n (%) | 80 (8.9) | 74 (8.2) | 2.29 | 0.674 |
| Hemoglobin, g/L, mean (SD) | 126.6 (22.0) | 128.3 (22.4) | 1.66 | 0.103 |
| **Risk of Gastrointestinal Bleeding** |  |  |  |  |
| Previous History of Peptic Ulcer or Gastrointestinal Bleeding, n (%) | 52 (5.8) | 54 (6.0) | 0.88 | 0.92 |
| Coagulopathy, n (%) | 15 (1.7) | 9 (1.0) | 5.32 | 0.304 |
| **Medication Use before Surgery** |  |  |  |  |
| Antibiotics, n (%) | 819 (90.8) | 842 (93.3) | 8.82 | 0.055 |
| RAASi, n (%) | 113 (12.5) | 119 (13.2) | 1.96 | 0.725 |
| NSAIDs, n (%) | 306 (33.9) | 285 (31.6) | 4.83 | 0.316 |
| Diuretics, n (%) | 120 (13.3) | 117 (13.0) | 0.91 | 0.889 |
| Contrast, n (%) | 55 (6.1) | 63 (7.0) | 3.55 | 0.505 |
| **Surgery Profiles** |  |  |  |  |
| Cardiac, n (%) | 39 (4.3) | 31 (3.4) | 4.35 | 0.394 |
| ASA Score≥3, n (%) | 362 (40.1) | 350 (38.8) | 2.69 | 0.596 |
| Emergent, n (%) | 83 (9.2) | 84 (9.3) | 0.36 | 1.000 |
| Intraoperative blood infusion, n (%) | 144 (16.0) | 135 (15.5) | 2.80 | 0.558 |
| **Outcomes** |  |  |  |  |
| Postoperative AKI | 62 (6.9) | 38 (4.2) | / | 0.018 |
| In-hospital AKI | 74 (8.2) | 45 (5.0) | / | 0.008 |
| In-hospital mortality | 6 (0.7) | 2 (0.2) | / | 0.288 |

Matching: demographic parameters (age, gender, and BMI), chronic comorbidities (hypertension, diabetes mellitus, cardiovascular disease, and cerebrovascular disease), baseline laboratory tests (eGFR, and hemoglobin), risk of gastrointestinal bleeding (previous history of peptic ulcer or gastrointestinal bleeding, and coagulopathy), medication use before surgery (antibiotics, RAASi, NSAIDs, diuretics, and contrast), and surgery profiles (cardiac surgery, emergent surgery, ASA score ≥ 3, and intraoperative blood infusion).

Abbreviation: BMI, body mass index; eGFR, estimated glomerular filtration rate; RAASi, renin-angiotensin-aldosterone system inhibitor; NSAIDs, non-steroidal anti-inflammatory drugs; ASA, American Society of Anesthesiologists.

**Supplementary Table 2. Sensitivity Analysis of the Association between PPI Usage before Major Surgeries and Postoperative AKI**

|  | **No. Patients with Event, n (%)** | | **Odds Ratio (95% Confidence Interval)** | |
| --- | --- | --- | --- | --- |
| **Outcome** | **PPI users, n (%)** | **PPI non-users, n (%)** | **Crude** | **Adjusted ^a^** |
| Postoperative AKI ^b^ | 62 (7.8) | 346 (1.7) | 4.85 (3.67, 6.42) | 1.61 (1.12, 2.32) |
| Postoperative AKI ^c^ | 72 (7.6) | 130 (1.3) | 6.50 (4.83, 8.73) | 1.75 (1.06, 2.89) |

a: Adjusted for demographic parameters (age, gender, and BMI), chronic comorbidities (hypertension, diabetes mellitus, cardiovascular disease, cerebrovascular disease, peripheral vascular disease, and chronic obstructive pulmonary disease), baseline laboratory tests (eGFR, and hemoglobin), risk of gastrointestinal bleeding (previous history of peptic ulcer or gastrointestinal bleeding, and coagulopathy), medication use before surgery (antibiotics, RAASi, NSAIDs, diuretics, and contrast), and surgery profiles (cardiac surgery, laparoscopy, emergent surgery, ASA score ≥ 3, duration of surgery ≥ 120 min, and intraoperative blood infusion).

b: Study population: A total of 405 patients with long-term indications for PPI use at admission were excluded, and the remaining 21,128 patients were included in the analysis.

c: Study population: A total of 10,233 patients who initiated PPI postoperatively were excluded, and the remaining 11,300 patients were included in the analysis.
